# Engaging Patients in Co-Design of Mobile Health Tools for Surgical Site Infection Surveillance: Implications for Research and Implementation

**DOI:** 10.1089/sur.2019.148

**Published:** 2019-09-10

**Authors:** Danielle C. Lavallee, Jenney R. Lee, John L. Semple, William B. Lober, Heather L. Evans

**Affiliations:** ^1^ Department of Surgery, University of Washington, Seattle, Washington. Seattle, Washington.; ^2^ University of Toronto, Women's College Hospital, Toronto, Ontario, Canada.; ^3^ Departments of Health Informatics and Global Health, University of Washington, Seattle, Washington. Seattle, Washington.; ^4^ Department of Surgery, Medical University of South Carolina, Charleston, South Carolina.

**Keywords:** mHealth, patient-centered care, patient engagement, patient-generated health data, surgical site infection

## Abstract

***Background:*** As the use of patient-owned devices, including smartphones and tablets, to manage day-to-day activities grows, so does healthcare industry's interest to better leverage technology to engage patients. For surgical care, a unique opportunity exists to capture patient-generated health data (PGHD) including photographs. As part of a broader initiative to evaluate PGHD for surgical site infection (SSI) surveillance, we sought evidence regarding patient involvement and experience with PGHD for SSI monitoring and surveillance.

***Methods:*** Through a scoping review of the literature and semi-structured stakeholder interviews we gathered evidence on what is currently known about patient perspectives of and experiences with mobile health (mHealth) interventions for post-operative recovery. We presented findings to and discussed with the ASSIST PGHD Stakeholder Advisory Group (PSAG) to generate priorities for further examination.

***Results:*** Our scoping review yielded 34 studies that addressed post-discharge use of PGHD for monitoring and surveillance of SSI. Of these, 16 studies addressed at least one outcome regarding patient experience; the most commonly measured outcome was patient satisfaction. Only three studies reported on patient involvement in the development of PGHD tools and interventions. We conducted interviews (n = 24) representing a range of stakeholder perspectives. Interviewees stressed the importance of patient involvement in tool and program design, noting patient involvement ensures the “work” that patients do in their daily lives to manage their health and healthcare is recognized. Discussion of evidence with the ASSIST PSAG resulted in formal recommendations for direct involvement of patients and caregivers for future work.

***Conclusions:*** While mHealth initiatives to advance post-operative management offer the ability to improve patient engagement, work is needed to ensure the patient voice is reflected. Active engagement with patients and caregivers in the development of new technology, the design of new workflows, and the conduct of research and evaluation ensures that the patient experiences and values are incorporated.

## Patient-Generated Health Data and Surgical Site Infection Surveillance

The evolution of mobile health (mHealth) to expand care coordination outside of the clinical encounter is recognized increasingly as a way to improve patient experience and health outcomes. Growth in both the availability and use of patient-owned devices, such as smartphones and tablets, to manage day-to-day activities including health are driving the healthcare industry to leverage technology as part of patient engagement strategies better. One recent example is the U.S. Food and Drug Administration's (FDA) announcement of a dedicated digital health unit within the FDA's medical device center as part of a “pre-certification” program for mobile apps [1]. This step toward recognizing the potential value of patient-generated health data (PGHD) has implications for surgical care, particularly in the ability to support post-operative management, care coordination, and monitoring for surgical site infections (SSI) [2–4].

The post-operative period is a vulnerable time for patients. Once discharged home, patients and care partners are responsible for ensuring proper care to surgical wounds and navigating the healing process. Under standard practice, post-operative follow-up occurs approximately 2 weeks post-discharge, a period during which patients face the greatest risk for surgical site infection [5,6]. Mobile health introduces an opportunity to improve the quality of surgical care both through enhanced data capture and patient-provider communication outside of traditional healthcare encounters. The ability to capture serial photographic images coupled with other biometric and patient-reported data presents a unique opportunity to monitor and engage with patients about surgical site healing and the quality of patient recovery [7].

Although mHealth presents new capabilities for managing the post-discharge phase of surgical care, it also presents challenges for practicing surgeons, healthcare organizations, and patients themselves in navigating new technology and new data in the context of care delivery. The current application of PGHD in clinical, research, and public health settings is characterized by wide variations in practice and uncertainty regarding how to best utilize these data to support clinical care and surveillance efforts. Because the transmission of PGHD is increasingly common in the post-operative care setting, systematic evaluation of the potential clinical, administrative, public health, and economic impacts is needed.

## PGHD and SSI Monitoring: Understanding the Current State of Patient Involvement

The overall aim of the ASSIST project's Health Technology Assessment (HTA) was to address the need for systematic study of current uses of PGHD and to better define how PGHD should be leveraged for SSI monitoring and surveillance across clinical, research, and public health settings [8]. To support this initiative, we sought evidence regarding patient involvement and experience with PGHD for SSI monitoring and surveillance. The process incorporated a stakeholder-driven, iterative approach to refine topic areas, gather and organize the available evidence, and develop a set of recommendations to guide future work in this domain. We sought to understand patient experience through a review of the literature, interviews with key informants, and discussions with the ASSIST PGHD Stakeholder Advisory Group (PSAG) (Table 1).

**Table 1.** Methods for Evaluating Patient Engagement in PGHD for SSI Surveillance

*Phase*	*Total included*	*Objective*
Literature review	13 articles	Understand what is currently known about patient experience
Key Informant interviews (phase 1)	21 interviews	Explore a variety of stakeholder perspectives on patient experience
Stakeholder advisory group meeting	25 attendees	Illuminate gaps in knowledge about patient experience and set priorities for future work
Key Informant interviews (phase 2)	3 interviews	Engage patients to understand their experiences, needs, and preferences

The first step of the process included a literature review to gather information on what is currently known about patient perspectives and experiences of mHealth interventions for post-operative recovery at home. Second, we conducted key informant interviews with a range of stakeholders (i.e., providers, informaticists, researchers, industry representatives) in the field of mHealth and PGHD for SSI detection and monitoring. We further engaged with stakeholders through convening a full-day meeting of a PGHD Stakeholder Advisory Group (PSAG), during which we presented initial findings of the ASSIST project and solicited feedback and generated priorities for further examination. These stakeholder engagement activities included input from clinicians, researchers, infection preventionists, design and ethics experts, and data scientists, among others. Stakeholders provided insights gained through working with patients in research and clinical settings. Through our stakeholder engagement activities during the initial round of key informant interviews and the PSAG workshop we were able to identify and speak directly with patients who had experience using mHealth apps for monitoring post-operative recovery following discharge from the hospital.

This final phase of interviews provided direct insight into the patient experience, and allowed us to explore patient perspectives on perceived benefits and potential drawbacks to the use of mHealth and PGHD and to elaborate on the value proposition for its use. Findings from our process are summarized below.

### Patient experience is important and understudied among peer-reviewed literature

Our literature search yielded 34 studies that addressed post-discharge use of PGHD for monitoring and surveillance of SSI. Of these, 16 studies addressed at least one element of the patient experience; the most commonly measured outcome associated with patient experience was patient satisfaction (Table 2). In general, studies reported high levels of patient satisfaction associated with use of PGHD for post-operative management [9–22]. Satisfaction was generally assessed via questionnaire or survey. Outcomes related to patient satisfaction with an intervention were (necessarily) assessed once the intervention concluded. Of note, no consistent measures for satisfaction or patient experience were used across studies. Only three studies reported on patient involvement in the development of PGHD tools and interventions [23–25]. These studies provide a unique view into the needs and perspectives that patients hold in relation to post-operative management. Specifically, these studies describe features patients desire as part of a tool to capture PGHD, challenges they foresee in their ability to follow a protocol for submitting PGHD, and needs in the post-operative period. These studies carried out the assessments prior to launch of the intervention with the intent that the findings would inform further iteration in tool or program development.

**Table 2.** Measures of Patient Experience

*Outcome*	*Number of articles*	*Measure used*
Satisfaction	14	Semi-structured interviews; survey questionnaire
Design factors	2	Semi-structured interviews
Usability	2	System Usability Score

### Key informants indicate patient engagement is underutilized

The first round of key informant interviews included 21 individuals representing a range of stakeholder perspectives. Interviews included designers, clinicians, vendors, data scientists, researchers, and infection preventionists with expertise in the use of PGHD for post-operative monitoring. Although patient key informants were sought out, we were unable to identify patients to participate in this phase of interviews. Reaching patient key informants was made difficult by the lack of a patient organization for SSI, or an easily identifiable patient population who had experienced SSI. However, many of the interviewees had direct experience working with patients in either clinical or research settings and reflected their insights about the patient experience from their own experiences working directly with patients. Interviewees stressed the importance of patient involvement in tool and program design, noting that programs seeking to leverage PGHD for post-operative monitoring by definition require active participation from patients, and that reliable data rely on reliable participation. Underlying this sentiment is the need to understand fully the patient value proposition for use of PGHD in the post-operative period.

The question of what motivates patients to fully engage in such programs, and what value they derive from their participation is one important element of the patient experience identified by key informants. Another is the need to evaluate what features of mHealth tools and programs may influence patient participation. Key informants noted that particular barriers and facilitators to patient participation exist. On the basis of their own experiences, key informants suggested that potential barriers might include lack of sufficient education/training on the use of mHealth apps, and the idea that surgical wounds may be cosmetically distressing to some patients. They also noted that patients may be more highly engaged if the app or tool includes a mechanism for them to receive feedback from providers (i.e., it allows bidirectional communication).

### Stakeholders support increased patient involvement

The findings from the literature review and key informant interviews were presented for discussion at the PSAG workshop. Facilitated discussion of findings revealed additional insights from stakeholders about the importance of patient involvement related to patient experience, design considerations, and implications for implementation. Stakeholders noted that patients increasingly submit photos and other symptom data to their care teams in an informal/unstructured capacity. This reinforces the concept that formalizing tools to capture and utilize PGHD in the post-operative setting is both desired by patients and needed by clinical teams. Previous work by some attendees included examination of the patient experience, which highlighted the need for training, the role of care partners (e.g., family members), and the importance of streamlining apps to facilitate patient ease of use.

“Patient work” was an area that stakeholders identified as needing additional examination. This concept is related to the fact that patients and providers may have diverging needs, and fully understanding those needs is key to accommodating the work that both groups do in carrying out tasks associated with generation and utilization of PGHD. Stakeholders also voiced the importance of understanding a diverse range of patient perspectives. This includes ensuring a broad range of surgical experiences, and those with little/no and a high degree of technological knowledge, as well as patients from a wide range of demographic backgrounds are represented. There was strong consensus from workshop attendees that additional key informant interviews with patients would make an important contribution to what is known about patient experience with using mHealth for post-operative management.

### Integrating patient experience with health and healthcare advances mHealth development and implementation

Through our work with stakeholders we identified additional key informants who agreed to be interviewed regarding the patient experience using mHealth for post-operative care coordination. The inclusion of these key informants was made possible through the relationships developed during stakeholder engagement work over the course of the project. These key informants had been participants in an intervention that used PGHD submitted via an mHealth app to monitor patients after breast surgery, including a nurse manager who had facilitated patient care during the program, as well as two patients. The findings from our initial work (literature review, first phase of interviews, and the PSAG workshop) informed the topics covered in these interviews. We asked interviewees about their experiences using an app to track post-operative recovery, including positive and negative aspects of participation, their experiences with post-operative complications (if any), and recommendations for future research and implementation efforts. Across the responses several notable patterns emerged.

First, interviewees reported that using the app to track their recovery was simple, and easy to carry out. Patients reported that they had no difficulty in taking photos or submitting answers to prompts. One patient noted that prior to surgery she was skeptical about using the app, thinking that recording the data would be “one more thing to worry about,” but had ultimately found it straightforward and rewarding to participate.

Second, interviewees reported increased convenience from using the app. Both patients reported that using the app had kept them from needing additional in-person visits with their surgeon. In one instance a patient noted that data she submitted via the app had alerted her provider to a developing infection, for which she was promptly issued a prescription for antibiotics, allowing her to avoid a trip to the clinic while still in a state of recovery. She described this experience as being “… so great, because had I had to come in that would have just been annoying; having to put something on, having to come [to the clinic], having to wait in a waiting room for so long, and for [the provider] to give me a script anyways.”

Third, interviewees expressed that using the app gave them a strong sense of reassurance that someone was watching over them in their recovery process. Patients expressed this as a sense of feeling connected. As one patient put it, “I felt like I could just relax more, and I knew that it would be picked up if anything was going on.” When probed about any negative aspects of participation patients did not report any drawbacks or barriers. Recommendations included expanding similar programs to include other surgical procedures and patient populations, in particular to patients who live far away from the hospital where their surgery occurs.

## Where Do We Go from Here: Involving Patients in Co-Design, Research, and Implementation

**FIG. 1.**
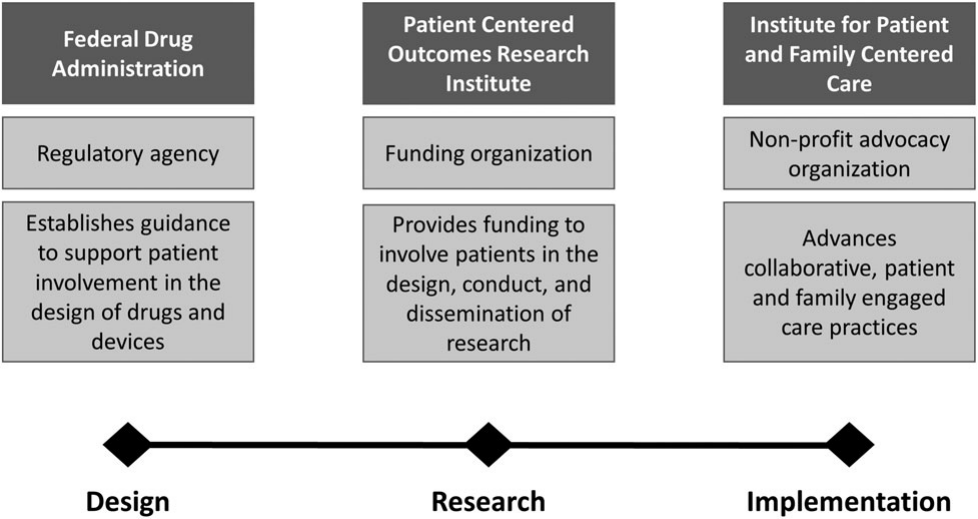
Organizations that promote patient engagement.

The success of mHealth in the context of post-operative surgical care and SSI surveillance hinges on the involvement of patients in the co-design of new technology, in the conduct of research, and the design and evaluation of implementation efforts in clinical care. Patient involvement ensures the “work” that patients do in their daily lives to manage their health and healthcare is recognized. This is often invisible from a design and medical system perspective, yet directly influences how patients engage in care/with new tools [26,27]. The context of this work can be informed through other national initiatives to promote patient involvement in product development, research, and healthcare delivery (Fig. 1).

### Patient-centered focus in regulatory decisions

The FDA is promoting the advancement of the patient voice in regulatory decisions. Recently published draft guidance provides researchers and industry sponsors insight on how patients can inform drug development and device design [28]. Although most mHealth products for SSI surveillance will not undergo FDA certification, the FDA's newly released guidance on involving patients in development of medical devices provides a useful framework for identifying what elements of the patient experience should be understood and how that can guide development. It recognizes the importance of engaging patients and care partners with lived experience to better understand and incorporate insight on how the condition impacts health and health related quality of life.

### Advancing patient-centered outcomes research

The direct involvement of patients and healthcare stakeholders in the design, conduct, and dissemination of research findings seeks to ensure that research funding aligns with the needs of healthcare decision-makers. The Patient Centered Outcomes Research Institute (PCORI) has played a substantial role in transforming the culture of research [29]. As a funding organization, for example, PCORI places patient and stakeholder engagement as a central requirement. In this context, patients and healthcare stakeholders serve as advisors to research, partner on research teams, and participate in research studies. To support this model for research, PCORI has developed tools and resources for the research and patient community to better support and facilitate partnerships. This includes a Patient Engagement Rubric as well as funding mechanisms to support capacity and training for research partnerships to form.

### Designing patient-centered care

Achieving patient-centered care and healthcare transformation requires that patients are involved in the design of care pathways. The Institute for Patient and Family-Centered Care (IPFCC), a non-profit organization, seeks to advance the practice of patient- and family-engaged care [30]. To this end, the organization creates tools and resources that promote and support collaborative partnerships among patients, families, and healthcare professionals across diverse healthcare settings. This includes providing guidance to health systems on establishing and sustaining patient and family advisory councils as part of organizational infrastructure. Care coordination, especially in the context of post-operative care, provides an ideal environment for partnering with patients and families to ensure safe, efficient, and respectful transitions in care.

## Conclusion

Patient involvement needs to be a central focus for mHealth initiatives to advance post-operative management as it offers the opportunity to improve care coordination and communication with patients. Although SSI surveillance is one important outcome from this work, this goal is only realized when the technology acknowledges and accounts for the work performed by patients outside the healthcare setting. This recognizes that collection and reporting of data by patients entails an additional burden on time and energy during an important time of recovery. Active engagement with patients and care partners in the development of new technology, the design of new workflows, and the conduct of research and evaluation ensures that the patient experiences and values are recognized and incorporated. In this manner, the products that are developed and the evidence that is generated will better reflect the needs of patients' needs in the post-operative setting.
